# TriPCE: A Novel Tri-Clustering Algorithm for Identifying Pan-Cancer Epigenetic Patterns

**DOI:** 10.3389/fgene.2019.01298

**Published:** 2020-01-15

**Authors:** Yanglan Gan, Ning Li, Yongchang Xin, Guobing Zou

**Affiliations:** ^1^ School of Computer Science and Technology, Donghua University, Shanghai, China; ^2^ School of Computer Engineering and Science, Shanghai University, Shanghai, China

**Keywords:** epigenetic analysis, pattern discovery, tri-clustering, FP-growth algorithm, pan-cancer

## Abstract

Epigenetic alteration is a fundamental characteristic of nearly all human cancers. Tumor cells not only harbor genetic alterations, but also are regulated by diverse epigenetic modifications. Identification of epigenetic similarities across different cancer types is beneficial for the discovery of treatments that can be extended to different cancers. Nowadays, abundant epigenetic modification profiles have provided a great opportunity to achieve this goal. Here, we proposed a new approach TriPCE, introducing tri-clustering strategy to integrative pan-cancer epigenomic analysis. The method is able to identify coherent patterns of various epigenetic modifications across different cancer types. To validate its capability, we applied the proposed TriPCE to analyze six important epigenetic marks among seven cancer types, and identified significant cross-cancer epigenetic similarities. These results suggest that specific epigenetic patterns indeed exist among these investigated cancers. Furthermore, the gene functional analysis performed on the associated gene sets demonstrates strong relevance with cancer development and reveals consistent risk tendency among these investigated cancer types.

## Introduction

Cancer genetics and epigenetics are closely linked in driving the cancer phenotype (Bailey et al., 2018). The vast majority of human cancers emerge from a gradual accumulation of somatic alterations and epigenetic abnormalities, which together lead to the malignant growth (Jones et al., 2016). Epigenetic changes can further enable tumor cells to escape from host immune surveillance and various treatments (You and Jones, 2012). Epigenetic abnormalities are usually observed as disrupted DNA methylation patterns (Chiappinelli et al., 2015), abnormal histone post translational modifications (Sawan and Herceg, 2010), and aberrant changes in chromatin organization (Allis and Jenuwein, 2016). How to identify epigenetic modification patterns that lead to the corresponding dysregulation in diverse cancers has become a critical research issue of cancer studies (Dawson, 2017; Kelly and Issa, 2017).

Great advancements have been made in delineating the underlying mechanisms of human cancers (Lawrence et al., 2014; Martincorena and Campbell, 2015). Extensive research has centered on the genetic aspect of cancers, such as how mutational activation and inactivation of cancer genes influence the cellular pathways (Vogelstein et al., 2013; Waddell et al., 2015). Recently, an increasing emphasis of drug discovery efforts has been targeting on the cancer epigenome (Flavahan et al., 2017). Many epigenome mapping projects have been gradually founded. The Cancer Genome Atlas Network (TCGA), BLUEPRINT, and the International Cancer Genome Consortium (ICGC) define the genome-wide distribution of epigenetic marks in many normal and cancerous tissues (Beck et al., 2012; Kundaje et al., 2015; Weinstein et al., 2015). Given the genome-wide distribution of epigenetic modifications of different cancers, it is urgent to decipher common epigenetic patterns across cancers and to understand the underlying mechanisms of tumorigenesis. Key epigenomic similarities shared by different cancer types would present an important opportunity to design effective cancer treatment strategies among cancers regardless of tissue or organ and enable the extension of effective treatments from one cancer type to another (Karlic et al., 2010; Gan et al., 2018).

To detect significant epigenetic patterns, existing computational methods mainly focus on identifying combinatorial states of different epigenetic marks. Specifically, CoSBI captures diverse histone modification patterns based on the correlations of different histone signals (Ucar et al., 2011). ChromHMM and HiHMM both apply a HMM model to annotate genomic sequences by the co-occurrence of multiple epigenetic marks (Ernst et al., 2011; Sohn et al., 2015). RFECS is developed mainly based on random forests (Rajagopal et al., 2013). IDEAS is able to jointly characterize epigenetic landscapes in many cell types and detect differential regulatory regions (Zhang et al., 2016). These methods have successfully identified the combinatorial epigenetic pattern in specific cell type. However, the relations among different cancer types still need to be investigated. Because DNA methylation in cancers has been addressed elsewhere (Kretzmer et al., 2015; Yang et al., 2016), here we only focus on the critical covalent histone modifications that are altered in various cancers, particularly the well-studied acetylation and methylation modifications.

In this paper, we proposed a tri-clustering approach, named TriPCE, for integrative pan-cancer epigenomic analysis. The method TriPCE adopts a tri-clustering strategy to identify the coherent patterns of various epigenetic modifications across different cancer types. We applied TriPCE to investigate six critical epigenetic marks among seven cancer types, and identified significant pan-cancer epigenetic modification patterns. The results reveal that there exists consistent epigenetic modification tendency among these cancer types. Meanwhile, the gene function analysis demonstrates that these associated genes are strongly relevant with the cancer cellular pathway.

## Materials and Methods

### Datasets

To detect epigenetic similarities among different cancers, we analyzed the epigenome maps of seven cancer types, including A549, K562, HepG2, HCT116, Hela-S3, multiple myeloma-Cell Line, and sporadic Burkitt lymphoma-Cell Line. For the epigenetic marks, we first filtered out those marks that are not included in these seven cancer types, and then focused on six widely studied ones, including H3K4me1, H3K4me3, H3K9me3, H3K27ac, H3K27me3, and H3K36me3. Meanwhile, the RNA expression profiles of these cancers were also collected. Totally, we obtained 42 epigenome maps and 7 RNA expression profiles for these cancers. The datasets were downloaded from the website of NIH Roadmap Epigenome Project.

### General Scheme of the TriPCE Approach

**Figure 1:**
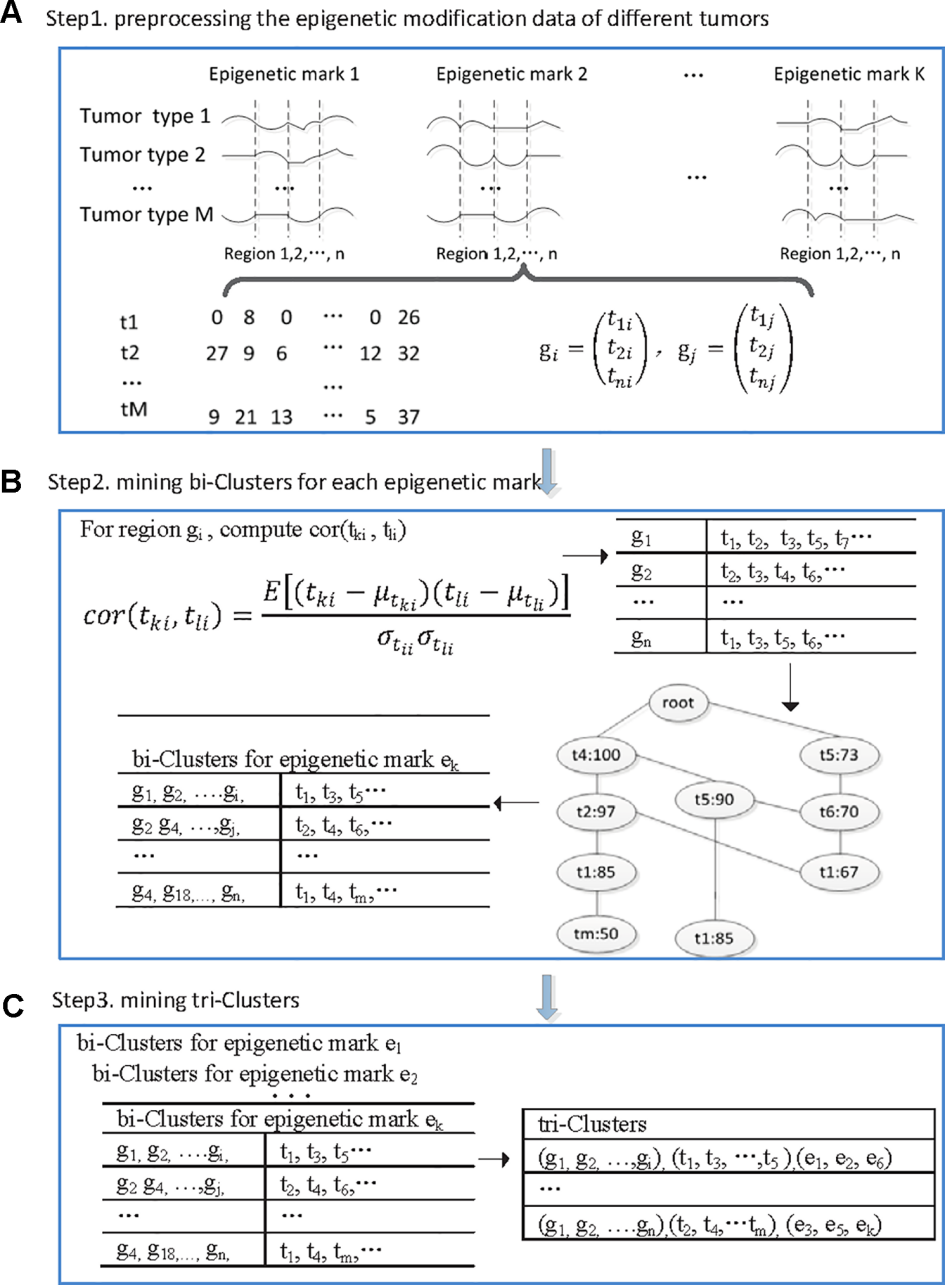
The flowchart of the proposed TriPCE approach. **(A)** Preprocessing the epigenetic modification data of different cancer types. **(B)** For each epigenetic mark, identifying bi-Clusters based on the FP-growth algorithm. **(C)** Mining tri-Clusters with coherent epigenetic modification patterns across different cancer types.

We developed a tri-clustering approach TriPCE to dissect the pan-cancer epigenetic pattern. The method not only explicitly detects combinatorial states of various epigenetic marks in different genomic segments, but also mines similar epigenetic patterns across different cancer types. The proposed TriPCE model has three key components, as shown in **Figure 1**. Firstly, preprocess the modification data of various epigenetic marks in different cancer types. Secondly, identify bi-Clusters based on FP-growth algorithm for each epigenetic mark. Thirdly, mine tri-Clusters with coherent epigenetic modification patterns across different cancer types.

**Step 1.** Preprocess the epigenetic modification data of different cancer types. Firstly, the genome was divided into consecutive genomic segments, with a typical segment size of 200 bps (Gan et al., 2017). For each epigenetic modification map, we computed the summary tag count of every segment. Then, each segment is associated with the intensities of a set of epigenetic modifications in each cancer type. To deduce the impact of the noise resulting from spurious tag counts in the ChIP-seq experiments, raw sequence read counts of each epigenetic modification were further normalized by the total number of reads followed by arcsine transformation (Pinello et al., 2014). Finally, according to the genome annotation data, the epigenetic distribution in the promoter regions was extracted.

After the preprocessing step, we gained six epigenetic profiles of seven cancer types along the promoter regions. Let *G* = {*ɡ*_1_, *ɡ*_2_,…, *ɡ_n_*} be a set of *n* genes, let *T* = {*t*_1_, *t*_2_,…, *t*_7_} be the investigated seven cancer types and let *E* = {*e*_1_, *e*_2_,…, *e*_6_} be the six epigenetic marks. For each epigenetic mark, the epigenetic profiles of different cancer types in the promoter regions of these genes are organized as a matrix $D_{k}=T\times G=\{t_{i,j}^{k}\}$ (with *i* ∈[1,2…,7], *j* ∈[1,2…, *n*], *k* ∈[1,2…,6]), where rows correspond to the cancer types, and columns correspond to those genes, respectively. Each entry $t_{i,j}^{k}$ is a vector representing the epigenetic profile of *e_k_* in the *i*th cancer along the promoter region of gene *j*.

**Step 2.** Identify bi-clusters based on FP-growth algorithm for each epigenetic mark. Given the preprocessed and reorganized epigenetic modification data matrix of each epigenetic mark, we first computed the Pearson correlation coefficients between the epigenetic profiles of any two cancer types at every promoter region, and then obtained a correlation coefficient matrix.

Specifically, for the promoter region *ɡ_i_,* we computed the Pearson correlation coefficients among the epigenetic modification distribution vectors of any different cancer types. If the calculated correlation coefficient is higher than a given threshold, the epigenetic modification trend in these two cancer types is regarded as coherent in this promoter region. Then, we added this cancer type to the corresponding itemset, which contains all the cancer types exhibiting similar epigenetic patterns in this region. Based on extensive experimental comparison, when the correlation coefficient threshold is set as 0.7, the identified epigenetic patterns are obviously coherent. For each epigenetic mark, we respectively constructed the corresponding similar itemsets for all promoter regions.

Based on the resulted itemset, we further identified the significant coherent epigenetic patterns using FP-growth algorithm (Han et al., 2004). FP-growth algorithm is a data mining method that was originally developed for frequent itemset mining in market basket analysis. Here, we adopted the FP-tree model to represent in a compact way all the cancer types with similar epigenetic patterns in different promoter regions. Then, it can be used to mine potential frequent itemsets and filter out most of the unrelated data. In this context, a typical frequent itemset represents a group of cancer types that share similar epigenetic patterns in abundant promoter regions. To gain the significant epigenetic states, we set the minimum support of genes as 10% of the investigated genes. For each frequent itemset, we then inversely identified the corresponding gene set and gained the bi-Cluster. The resulted bi-Cluster is in the form (“genomic regions,” “cancer types”), representing the cancer types exhibit similar epigenetic patterns in these genes. Similarly, we obtained the corresponding bi-Cluster sets for all investigated epigenetic marks.

**Step 3.** Mine tri-Clusters with coherent epigenetic modification patterns across different cancer types. After obtaining the bi-Cluster sets for each epigenetic mark, we further mined the tri-Clusters. By enumerating the maximum subsets of different epigenetic marks, we obtained the tri-Clusters. In detail, we respectively computed the intersection of the bi-Cluster sets from two epigenetic marks *e_k_* and *e_l_*, which are kept with the epigenetic marks to get possible tri-Clusters. Further, by filtering out the candidates with the support lower than the predefined minimum support, we obtained the significant tri-Clusters. Iteratively, we continued the process with another epigenetic mark until all the epigenetic marks were analyzed. We tried all such paths and kept the maximal tri-Clusters only. Each tri-Cluster is represented as (“genomic regions,” “cancer types,” “epigenetic marks”), listing a gene set with similar trend of epigenetic modifications in different cancer types. The resulted tri-Clusters indicate that the conserved epigenetic signatures in these genomic regions are shared by multiple cancer types.

### Functional Analysis of the Genes

From the identified tri-Clusters, we can obtain the gene sets associated with specific coherent epigenetic patterns. To investigate the potential functions of these genes, we performed the gene ontology (GO) enrichment analysis and pathway enrichment analysis *via* DAVID bioinformatics resources (Huang et al., 2007). The significant enrichment lists were obtained with P-value < 0.005.

## Results

### Identifying Similar Epigenetic Patterns Across Different Cancer Types

**Figure 2:**
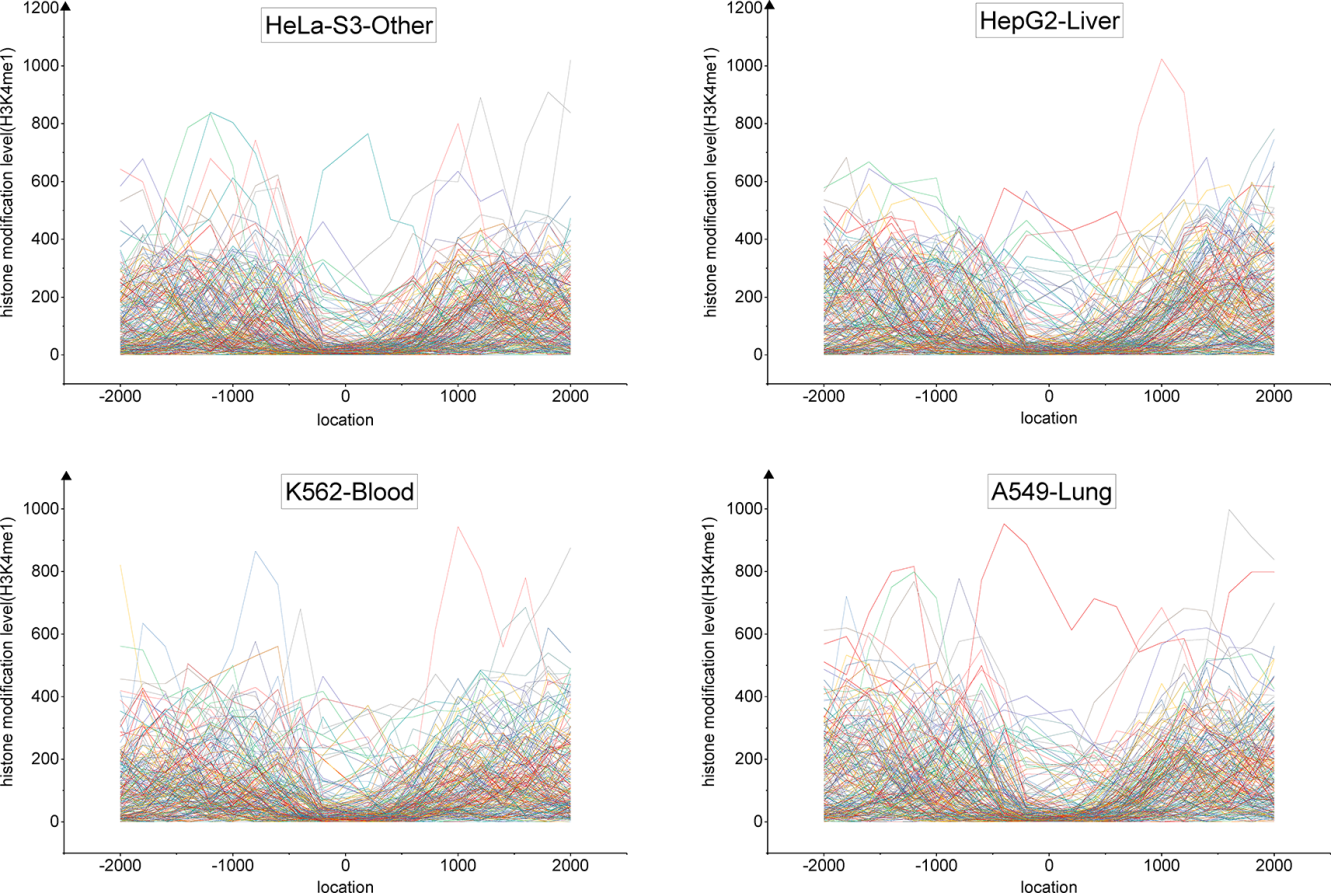
The profiles of epigenetic mark H3K4me3 in a typical bi-Cluster exhibit a similar pattern in four cancer types, including Hela-S3, HepG2, K562 and A549.

We developed a tri-clustering approach, TriPCE, to capture similar epigenetic patterns among different cancer types. TriPCE was applied to the genome-wide epigenetic modification maps of seven cancer types, including A549, K562, HepG2, HCT116, Hela-S3, multiple myeloma-Cell Line, and sporadic Burkitt lymphoma-Cell Line. For each epigenetic mark, TriPCE first groups the promoter regions based on the epigenetic modification profiles among different cancer types. **Figure 2** shows a typical bi-Cluster of epigenetic mark H3K4me1, which contains abundant genes with similar modification pattern in four cancer types, including Hela-S3, HepG2, K562, and A549. From this figure, we observe that the epigenetic profiles of these genes are similar in these cancer types. Then, the epigenetic profile shared by a cluster of promoter regions in multiple cancer types is considered to be an epigenetic pattern. Meanwhile, different cancer types share similar epigenetic patterns. This result is consistent with previous finding that H3K9me3/me2 and H3K36me3/me2 frequently observed in breast cancer (Liu et al., 2009), esophageal cancer (Yang et al., 2000), MALT lymphoma (Vinatzer et al., 2008), and lung sarcomatoid carcinoma (Italiano et al., 2006). Based on the identified bi-Clusters of these investigated epigenetic marks, we noted that cancers (HepG2 and HCT116) are clustered together and share a larger number of epigenetic marks, implying that they share more similar epigenetic regulation mechanisms.

**Figure 3:**
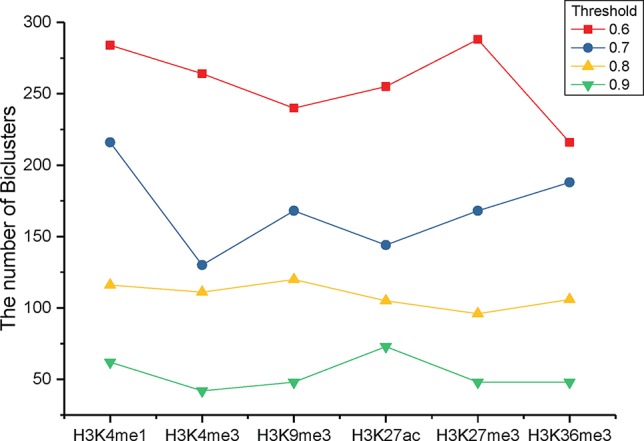
The numbers of bi-Clusters with varied similarity thresholds for different epigenetic marks.

To identify the significant modification patterns, we set the minimal support of genes as 10% of the investigated genes. With diverse correlation coefficient thresholds, we respectively gained different numbers of bi-Clusters for epigenetic marks H3K4me1, H3K4me3, H3K9me3, H3K27me3, H3K36me3, and H3K27ac, among these cancer types, as shown in **Figure 3**. The comparison indicates that the similarities of these epigenetic marks are quite different. Under different threshold settings, the epigenetic mark H3K4me3 has a relatively small number of bi-Clusters, indicating that its profiles are less conserved and exhibit more variable patterns among these cancer types than other epigenetic marks. On the contrary, there are more similar epigenetic patterns of H3K4me1 and H3K27me3 among different cancer types (Baylin and Jones, 2016). The plasticity of epigenome depends on diverse environmental factors. Thus, it is not surprising that epigenotypes contribute to developmental human disorders and adult diseases (Brien et al., 2016). As the minimal support threshold slightly affects the trend among different epigenetic marks, we chose the bi-Clusters with threshold 0.7 for further analysis.

### Identifying Coherent Patterns Among Different Epigenetic Marks

From the above results, we notice that there are obvious differences among the investigated epigenetic modifications. To identify the conserved epigenetic states and explore the similar patterns of these epigenetic modifications, we further clustered these epigenetic marks based on the detected bi-Clusters. By systematically computing the intersection of the bi-Cluster sets from different epigenetic marks, we kept the tri-Clusters with the support higher than the predefined minimum support. The identified tri-Clusters are represented as triples (“genomic regions,” “cancer types,” “epigenetic marks”). Each tri-Cluster represents that the promoter region of these genes exhibits similar epigenetic modification patterns in the related cancer types.

**Figure 4:**
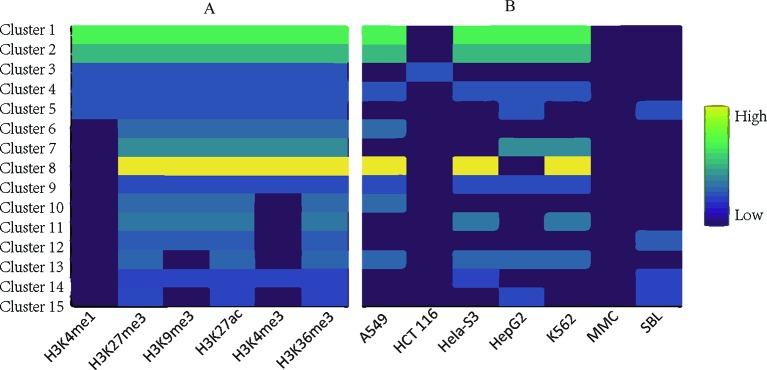
Typical epigenetic tri-Clusters. **(A)** The epigenetic marks (column) in each cluster (row). **(B)** The cancer types (column) in each cluster (row). Fold enrichment was calculated as the ratio between the number of genes in the tri-Cluster to that of all genes.

Applying TriPCE to the data set, we initially obtained 175 significant tri-Clusters. **Figure 4** shows the information of 15 typical clusters, including the epigenetic marks, the cancer types, and the supports of these tri-Clusters. The results indicate that specific genomic regions indeed share combinatorial epigenetic patterns across different cancer types. For example, the changing pattern of epigenetic modifications (H3K4me3, H3K9me3, H3K27me3, and H3K36me3) are shared by a large number of genes in cancer types A549, HepG2, and K562. On the contrary, some epigenetic modification patterns are only coherent in certain cancer types. Among these resulted clusters, we observe that the similar patterns of H3K36me3, H3K27ac, and H3kK27me3 exist in fewer cancer types, such as HepG2 and sporadic Burkitt lymphoma-Cell Line. Notably, these identified tri-Clusters reveal more information about the epigenetic patterns among these cancer types.

### Analyzing the Potential Roles of Associated Genes

Based on the detected tri-Clusters, we further obtained those gene sets that exhibit coherent epigenetic patterns in different cancer types. Previous studies have shown that the modification intensities are significantly distinct between high-expression gene promoters and low-expression gene promoters, which suggests that these chromatin components have significant effect on gene regulation (Su et al., 2012). To investigate the potential functions of those genes in the cellular control pathways, we performed a systematic GO enrichment analysis using DAVID tools (https://david.ncifcrf.gov/). Then, for the associated gene sets in the identified tri-Clusters, we respectively summarized the key biological processes and pathways that they are involved in.

Overall, we found that those genes enriched in tri-Clusters exhibit an enrichment for cancer-related functions. **Table 1** lists the significant GO terms of a typical tri-Cluster (P-value < 0.005). In this tri-Cluster, the genes exhibit coherent modification patterns on epigenetic marks (H3K4me1, H3K4me3, H3K9me3, H3K27ac, and H3K27me3) in cancer types (HeLa-S3, HepG2, multiple myeloma-Cell Line, and sporadic Burkitt lymphoma-Cell Line). In the table, terms “positive regulation of cell proliferation” and “negative regulation of apoptotic process” are enriched in these gene sets. This result implies that the identified genes in this tri-Cluster are essential for cell proliferation and apoptotic process, which has been reported to be related to cancer development by previous researches (Deng et al., 2016). Meanwhile, the term “positive regulation of gene expression” is also enriched in the gene set, further indicating that these genes might perform important regulation roles in these cancers.

**Table 1:** Functional enrichment of genes in the identified tri-Clusters.

Term type	Term name	P-value	Term type	Term name	P-value
BP	Positive regulation of cell proliferation	2.84E-06	MF	Protein binding	1.10E-12
BP	Translational initiation	1.18E-05	MF	Poly(A) RNA binding	3.90E-10
BP	mRNA processing	2.72E-05	MF	RNA binding	2.13E-05
BP	Cell division	4.08E-05	MF	Glutathione binding	7.85E-04
BP	rRNA processing	2.70E-04	MF	Enzyme regulator activity	4.02E-03
BP	RNA splicing	4.04E-04	MF	Nucleosomal DNA binding	4.25E-03
BP	Positive regulation of gene expression, epigenetic	9.41E-04	MF	Translation initiation factor activity	4.30E-03
BP	Protein targeting to Golgi	8.87E-05	MF	Glutathione transferase activity	8.00E-03
BP	Nitrobenzene metabolic process	1.14E-04	MF	Protein binding, bridging	4.33E-03
BP	Xenobiotic catabolic process	1.13E-03	MF	ATP binding	4.57E-03
BP	mRNA splicing, *via* spliceosome	1.14E-03	CC	Nucleoplasm	6.18E-13
BP	Sister chromatid cohesion	2.13E-03	CC	Cytosol	3.96E-07
BP	SRP-dependent cotranslational protein targeting to membrane	1.06E-03	CC	Membrane	7.68E-06
BP	Negative regulation of transcription, DNA-templated	1.55E-03	CC	Nucleus	2.34E-04
BP	Negative regulation of apoptotic process	1.88E-03	CC	Cytoplasm	2.69E-04
BP	Nucleosome assembly	3.86E-03	KEGG	Glutathione metabolism	1.09E-03
BP	Glutathione derivative biosynthetic process	4.18E-03	KEGG	Systemic lupus erythematosus	1.93E-03

## Discussion

Identifying epigenetic patterns is important to understand epigenetic mechanisms in various cancers. The detected patterns among different cancers could demonstrate critical cross-cancer similarities, which reveals some consistent clinical risk among different cancer types and further suggests strong clinical relevance. Our knowledge about the patterns of epigenetic modifications and the cause and consequence of them is still limited. Computational approach that exploits the complex epigenomic landscapes and discovers significant signatures out of them is required. Previous computational methods for analyzing epigenomes primarily focus on the combinatorial states of different epigenetic marks in a specific cell type. Differently, we developed a tri-clustering approach TriPCE for integrative pan-cancer epigenomic analysis. Based on the FP-tree structure, TriPCE can compactly represent all similar cancer types in the promoter regions for a specific epigenetic mark. Using the constructed FP-tree, the frequent patterns are then detected to yield the set of bi-Clusters of this epigenetic mark, indicating the similar epigenetic pattern in these cancer types along these genomic regions. TriPCE further mines the final tri-Clusters based on the bi-Clusters of all investigated epigenetic marks, explicitly detecting combinatorial epigenetic states in different genomic segments and similar epigenetic changes across different cancer types. In the proposed approach TriPCE, the tri-Cluster enumeration is an expensive operation. In the future we plan to develop heuristic techniques to efficiently prune the search space, and then improve the efficiency of mining the tri-Clusters. We applied TriPCE to uncover the similar patterns of six epigenetic marks among seven cancer types and successfully identified significant cross-cancer epigenetic modification similarities, which suggests that there exhibits consistent epigenetic modification tendency among these investigated cancer types. Furthermore, the gene functional analysis demonstrates that these associated genes are strongly relevant with the cancer cellular pathway.

## Data Availability Statement

All datasets generated for this study are included in the article/supplementary material.

## Author Contributions

YG is responsible for the main idea, as well as the completion of the manuscript. NL and YX have developed the algorithm and performed data analysis. GZ has coordinated data preprocessing and supervised the effort. All authors have read and approved the final manuscript.

## Funding

This work and the publication costs were supported in part by the National Natural Science Foundation of China (61772128, 61772367), National Key Research and Development Program of China (2016YFC0901704), Shanghai Natural Science Foundation (17ZR1400200,18ZR1414400), and the Fundamental Research Funds for the Central Universities (2232016A3-05),

## Conflict of Interest

The authors declare that the research was conducted in the absence of any commercial or financial relationships that could be construed as a potential conflict of interest.

## References

[B1] Allis C. D. Jenuwein T. (2016). The molecular hallmarks of epigenetic control. Nat. Rev. Genet. 17, 487. 10.1038/nrg.2016.59 27346641

[B2] Bailey M. H. Tokheim C. Porta-Pardo E. Sengupta S. Bertrand D. Weerasinghe A. (2018). Comprehensive characterization of cancer driver genes and mutations. Cell 173, 371–385. 10.1016/j.cell.2018.02.060 29625053 PMC6029450

[B3] Baylin S. B. Jones P. A. (2016). Epigenetic determinants of cancer. Cold Spring Harbor Perspect. In Biol. 8, a019505. 10.1101/cshperspect.a019505 PMC5008069 27194046

[B4] Beck S. Bernstein B. E. Campbell R. M. Costello J. F. Dhanak D. Ecker J. R. (2012). A blueprint for an international cancer epigenome consortium. a report from the aacr cancer epigenome task force. Cancer Res. 72, 6319–6324. 10.1158/0008-5472.CAN-12-3658 23188507

[B5] Brien G. L. Valerio D. G. Armstrong S. A. (2016). Exploiting the epigenome to control cancer-promoting gene-expression programs. Cancer Cell 29, 464–476. 10.1016/j.ccell.2016.03.007 27070701 PMC4889129

[B6] Chiappinelli K. B. Strissel P. L. Desrichard A. Li H. Henke C. Akman B. (2015). Inhibiting dna methylation causes an interferon response in cancer *via* dsrna including endogenous retroviruses. Cell 162, 974–986. 10.1016/j.cell.2015.07.011 26317466 PMC4556003

[B7] Dawson M. A. (2017). The cancer epigenome: Concepts, challenges, and therapeutic opportunities. Science 355, 1147–1152. 10.1126/science.aam7304 28302822

[B8] Deng S. P. Zhu L. Huang D. S. (2016). Predicting hub genes associated with cervical cancer through gene co-expression networks. IEEE/ACM Trans. Comput. Biol. Bioinf. 13, 27–35. 10.1109/TCBB.2015.2476790 26415208

[B9] Ernst J. Kheradpour P. Mikkelsen T. S. Shoresh N. Ward L. D. Epstein C. B. (2011). Mapping and analysis of chromatin state dynamics in nine human cell types. Nature 473, 43. 10.1038/nature09906 21441907 PMC3088773

[B10] Flavahan W. A. Gaskell E. Bernstein B. E. (2017). Epigenetic plasticity and the hallmarks of cancer. Science 357, eaal 2380. 10.1126/science.aal23800.1126/science.aal2380 PMC5940341 28729483

[B12] Gan Y. Tao H. Guan J. Zhou S. (2017). ihms: a database integrating human histone modification data across developmental stages and tissues. BMC Bioinf. 18, 103. 10.1186/s12859-017-1461-y PMC5303264 28187703

[B11] Gan Y. Dong Z. Zhang X. Zou G. (2018). “Tri-clustering analysis for dissecting epigenetic patterns across multiple cancer types,” in International Conference on Intelligent Computing (Springer), 330–336.

[B13] Han J. Pei J. Yin Y. Mao R. (2004). Mining frequent patterns without candidate generation: A frequent-pattern tree approach. Data Min. knowl. Discovery 8, 53–87. 10.1023/B:DAMI.0000005258.31418.83

[B14] Huang D. W. Sherman B. T. Tan Q. Kir J. Liu D. Bryant D. (2007). David bioinformatics resources: expanded annotation database and novel algorithms to better extract biology from large gene lists. Nucleic Acids Res. 35, W169–W175. 10.1093/nar/gkm415 17576678 PMC1933169

[B15] Italiano A. Attias R. Aurias A. Pérot G. Burel-Vandenbos F. Otto J.(2006). Molecular cytogenetic characterization of a metastatic lungsarcomatoid carcinoma: 9p23 neocentromere and 9p23 p24 amplification including jak2 and jmjd2c. Cancer Genet. Cytogenet. 167, 122–130. 10.1016/j.cancergencyto.2006.01.004 16737911

[B16] Jones P. A. Issa J. P. J. Baylin S. (2016). Targeting the cancer epigenome for therapy. Nat. Rev. Genet. 17, 630–641. 10.1038/nrg.2016.93 27629931

[B17] Karlic R. Chung H. R. Lasserre J. Vlahovicek K. Vingron M. (2010). Histone modification levels are predictive for geneexpression. Proc. Natl. Acad. Sci. U.S.A. 107, 2926–2931. 10.1073/pnas.0909344107 20133639 PMC2814872

[B18] Kelly A. D. Issa J.-P. J. (2017). The promise of epigenetic therapy: reprogramming the cancer epigenome. Curr. Opin. Genet. Dev. 42, 68–77. 10.1016/j.gde.2017.03.015 28412585

[B19] Kretzmer H. Bernhart S. H. Wang W. Haake A. Weniger M. A. Bergmann A. K. (2015). Dna-methylome analysis in burkitt and follicular lymphomas identifies differentially methylated regions linked to somatic mutation and transcriptional control. Nat. Genet. 47, 1316–1325. 10.1038/ng.3413 26437030 PMC5444523

[B20] Kundaje A. Meuleman W. Ernst J. Bilenky M. Yen A. Heravimoussavi A. (2015). Integrative analysis of 111 reference human epigenomes. Nature 518, 317–330. 10.1038/nature14248 25693563 PMC4530010

[B21] Lawrence M. S. Stojanov P. Mermel C. H. Robinson J. T. Garraway L. A. Golub T. R. (2014). Discovery and saturation analysis of cancer genes across 21 tumour types. Nature 505, 495. 10.1038/nature12912 24390350 PMC4048962

[B22] Liu G. Bollig-Fischer A. Kreike B. van de Vijver M. J. Abrams J. Ethier S. P. (2009). Genomic amplification and oncogenic properties of the gasc1 histone demethylase gene in breast cancer. Oncogene 28, 4491. 10.1038/onc.2009.297 19784073 PMC2795798

[B23] Martincorena I. Campbell P. J. (2015). Somatic mutation in cancer and normal cells. Science 349, 1483–1489. 10.1126/science.aab4082 26404825

[B24] Pinello L. Xu J. Orkin S. H. Yuan G. C.(2014). Analysis of chromatin-state plasticity identifiescell-type-specific regulators of h3k27me3 patterns. Proc. Natl. Acad. Sci. U.S.A. 111, E344. 10.1073/pnas.1322570111 24395799 PMC3903219

[B25] Rajagopal N. Xie W. Li Y. Wagner U. Wang W. Stamatoyannopoulos J. (2013). Rfecs: a random-forest based algorithm for enhancer identification from chromatin state. PloS Comput. Biol. 9, e1002968. 10.1371/journal.pcbi.1002968 23526891 PMC3597546

[B26] Sawan C. Herceg Z. (2010). Histone modifications and cancer. Adv. In Genet. 70, 57–85. 10.1016/B978-0-12-380866-0.60003-4 20920745

[B27] Sohn K.-A. Ho J. W. Djordjevic D. Jeong H.-h. Park P. J. Kim J. H. (2015). hihmm: Bayesian non-parametric joint inference of chromatin state maps. Bioinformatics 31, 2066–2074. 10.1093/bioinformatics/btv117 25725496 PMC4481846

[B28] Su J. Liu S. Wu X. Lv J. Liu H. Zhang R. (2012). Revealing epigenetic patterns in gene regulation through integrative analysis of epigenetic interaction network. Mol. Biol. Rep. 39, 1701–1712. 10.1007/s11033-011-0910-3 21625856

[B29] Ucar D. Hu Q. Tan K. (2011). Combinatorial chromatin modification patterns in the human genome revealed by subspace clustering. Nucleic Acids Res. 39, 4063–4075. 10.1093/nar/gkr016 21266477 PMC3105409

[B30] Vinatzer U. Gollinger M. Müllauer L. Raderer M. Chott A. Streubel B. (2008). Mucosa-associated lymphoid tissue lymphoma: novel translocations including rearrangements of odz2, jmjd2c, and cnn3. Clin. Cancer Res. 14, 6426–6431. 10.1158/1078-0432 18927281

[B31] Vogelstein B. Papadopoulos N. Velculescu V. E. Zhou S. Diaz L. A. Kinzler K. W. (2013). Cancer Genome Landsc. Science 339, 1546–1558. 10.1126/science.1235122 23539594 PMC3749880

[B32] Waddell N. Pajic M. Patch A.-M. Chang D. K. Kassahn K. S. Bailey P. (2015). Whole genomes redefine the mutational Landscape of pancreatic cancer. Nature 518, 495. 10.1038/nature14169 25719666 PMC4523082

[B33] Weinstein J. N. Collisson E. A. Mills G. B. Shaw K. R. Ozenberger B. A. Ellrott K. (2015). The cancer genome atlas pan-cancer analysis project. Nat. Genet. 45, 1113–1120. 10.1038/ng.2764 PMC3919969 24071849

[B35] Yang Z.-Q. Imoto I. Fukuda Y. Pimkhaokham A. Shimada Y. Imamura M. (2000). Identification of a novel gene, gasc1, within an amplicon at 9p23–24 frequently detected in esophageal cancer cell lines. Cancer Res. 60, 4735–4739. 10987278

[B34] Yang X. Lin G. Zhang S.(2016). Comparative pan-cancer dna methylation analysis reveals cancer common and specific patterns. Briefings Bioinf. 18, 761. 10.1093/bib/bbw063 27436122

[B36] You J. S. Jones P. A. (2012). Cancer genetics and epigenetics: Two sides of the same coin? Cancer Cell 22, 9. 10.1016/j.ccr.2012.06.008 22789535 PMC3396881

[B37] Zhang Y. An L. Yue F. Hardison R. C. (2016). Jointly characterizing epigenetic dynamics across multiple human cell types. Nucleic Acids Res. 44, 6721–6731. 10.1093/nar/gkw278 27095202 PMC5772166

